# Determining the clinical knowledge and practice of Australian podiatrists on children with developmental coordination disorder: a cross-sectional survey

**DOI:** 10.1186/s13047-019-0353-y

**Published:** 2019-08-13

**Authors:** Mitchell Smith, Helen A. Banwell, Emily Ward, Cylie M. Williams

**Affiliations:** 1 School of Health Sciences, University of South Australia, Adelaide, South Australia 5001 Australia; 2 International Centre for Allied Health Evidence, University of South Australia, Adelaide, South Australia 5001 Australia; 3 Allied Health, Peninsula Health, Frankston, Victoria 3199 Australia; 4 School of Primary and Allied Health, Monash University, Frankston, Victoria 3199 Australia

**Keywords:** Developmental coordination disorder, Children, Podiatry, Knowledge, Assessment, Management

## Abstract

**Background:**

Developmental coordination disorder (DCD) is a common condition in children affecting motor coordination. This impacts on academic performance, and activities of daily living. Literature surrounding interventions for DCD has focused mostly on physical and occupational therapies. However, it is known that children with DCD present to podiatrists as these children often also have abnormalities in lower limb functioning associated with the condition. This study aimed to determine current knowledge of Australian podiatrists regarding presentation, assessment, and management of children with developmental coordination disorder.

**Methods:**

A single-round survey, developed using SurveyMonkey®, was completed by a sample of Australian podiatrists. Data were collected through either online or paper means. Participants were asked about their familiarity with DCD and depending on their response, were directed via skip logic to questions on presentation, assessment and management strategies of DCD in children. Participants were also asked about their willingness and preferences for further education on DCD. Descriptive statistics were used to describe the data.

**Results:**

There were 365 Australian podiatrists who completed the survey. There were 30% (*n* = 109) who reported being familiar with DCD as a diagnosis, while a further 37% (*n* = 134) reported familiarity with alternate or outdated terminology associated with the DCD diagnosis. Participants who were familiar with DCD or terminology relating to DCD, showed good knowledge of signs and symptoms associated with DCD. Both familiar and unfamiliar participants favoured referral to other health professionals over completing assessments. Common podiatric management strategies such as footwear advice, orthoses, and strength training were the most frequently chosen by both groups, despite current evidence only supporting strength training as an intervention. Participants were willing to receive education on DCD through a range of both online and in-person mediums.

**Conclusion:**

The majority of Australian podiatrists were unfamiliar with DCD, despite its prevalence and symptomology falling within the podiatric scope. However, participants did overwhelmingly show willingness to receive further education on DCD. Further research should consider understanding the role of podiatrists in the assessment and management of children with DCD and the impact of the type of treatment strategies that may be provided.

**Electronic supplementary material:**

The online version of this article (10.1186/s13047-019-0353-y) contains supplementary material, which is available to authorized users.

## Background

Developmental coordination disorder (DCD) is a diagnosis made when children experience motor difficulties or dysfunction early in their development. These difficulties must significantly interfere with their daily life or academic achievement but cannot be attributed to another identifiable neurological condition or impairment [1]. DCD is frequently characterised by slow and awkward movement, commonly manifest in impaired ball skills, poor handwriting and other fine and gross motor skills disruption [2, 3]. Motor impairment in children with DCD greatly affects participation in common childhood activities such as using playground equipment and participation in organised sports. These activities are essential in children’s social development [4–6]. Consequently, children with DCD have also reported lower health-related quality of life, and more emotional disturbances than typically developing children [7–10].

Prevalence of DCD in Australia is estimated as 8% of children at age eight, although is generally accepted globally as 5–6% of school age children [11]. There is a reported higher proportion of males to females [11–13]. It is considered that many children who may fit the diagnostic criteria for DCD remain undiagnosed due to variations and ranges of signs and symptoms, as well as lack of awareness of the disorder [14].

Historically, physiotherapists and occupational therapists are the allied health professionals typically involved in assessment and management of children with DCD [11, 15]. Inclusion of other allied health professionals, such as podiatrists, in management of DCD symptomology has the potential to increase due to systems supporting people with disabilities, particularly in children and parents having greater say in who or what professions they seek support from when developing therapy goals. Clinical observations associated with DCD such as altered gait or difficulties with gross motor activities [16] makes this particularly relevant for podiatrists. These areas are those that podiatrists commonly have training or additional experience. There is limited research on the effectiveness of any podiatry-specific interventions for children with DCD, with only one published study investigating the use of foot orthoses [17]. This study found the use of foot orthoses to have a limited impact on spatiotemporal parameters of gait [17]. However, it is likely children with DCD are attending podiatry clinics due to parental concerns such as tripping, or simply ‘walking funny’. It may be within scope of practice for podiatrists to assess and manage the lower limb concerns of a child with DCD, and to understand when referral is appropriate when podiatrists have the appropriate skills, awareness or training. What is not known, is how these children are being assessed and managed by podiatrists, and the extent of the clinical knowledge and understanding of DCD amongst podiatrists in Australia.

The primary aim of this study was to determine the awareness and familiarity of Australian podiatrists with DCD or historic terminology for DCD. The secondary aims of the study were to capture current assessment and management strategies, and education preferences of podiatrists relating to DCD.

## Methodology

### Research design

This study was a quantitative, cross-sectional, survey design targeting all podiatrists in Australia. Ethical approval was gained from the University of South Australia Human Research Ethics Committee (Approval: 36556).

### Participants and settings

All registered Australian podiatrists were eligible to participate in this study (*n* = 4999 as of close of data collection in December 2017) [18]. A sample size calculation, based on *n =* 4999 and a 95% confidence interval and 5% margin of error, resulted in a required sample size of 357 participants to gain a representative sample of the profession [19]. The survey was advertised and disseminated through in-person engagement at local and national podiatry conferences and seminars, by email flyers, newsletters and online media (Facebook™ and Twitter™) and through the Australian Podiatry Council and state-based Podiatry Associations.

### Outcome measures

Data collection was via a single-round custom-developed, self-reported questionnaire using SurveyMonkey® [20], (Additional file 1). The survey contained two sections: demographic data and questions relating to participants’ clinical knowledge, experience, and education preferences concerning DCD.

Demographic data collected included years of clinical experience, gender (male, female, prefer not to say, unidentified), alma mater, highest qualification relating to podiatry, recency of practice, primary employment position as a podiatrist (private practice, public sector (community or hospital), and non-clinical (including research, education and administration positions)), state or territory of practice, average weekly hours worked and estimated proportion of paediatric clients as a percentage of overall clinical workload.

The measures used to determine participant clinical knowledge and experience were based on previous published work [21, 22] and developed further by the authors to highlight factors in presentation often assessed by podiatrists including muscle tone, gait, and foot posture, and to reflect common podiatric management options. The survey was piloted in two stages by six non-participating podiatrists with additional training in education, or extensive experience in working with children who have disorders affecting their movement. The final instrument asked participants to give their familiarity with the term DCD or alternatives (i.e. minimal motor dysfunction, minimal brain dysfunction, minimal cerebral dysfunction, clumsy child syndrome, and disorder of attention and motor planning (DAMP)) [3, 23, 24]. Those participants who were familiar with DCD were subsequently asked about their experiences with the clinical presentation, assessment and management techniques for children with DCD. Participants were given the options of the following commonly known presentations: low muscle tone, tripping, tiring easily, musculoskeletal pain, pes planus foot posture, ligamentous laxity, toe walking, delay in fine and gross motor skills, impaired proprioception, and poor motor planning, and presentations not commonly associated with DCD including skin changes, tibial torsion and metatarsus adductus to act as question distractors [2, 3, 11, 14, 16].

Participants who reported no familiarity with DCD were given a definition of the condition. This definition was:

*“Developmental coordination disorder (DCD) is a diagnosis given to children who present with motor difficulties or dysfunction which interferes with their daily life or academic achievement but cannot be attributed to another identifiable neurological condition such as cerebral palsy. It is estimated to affect approximately 5-8% of children. Common symptoms include poor coordination, low muscle tone, tripping, tiring easily, delay in both fine and gross motor skills, and difficulty following instructions”.*

Based on their understanding of this definition, participants were asked if they believed podiatrists could have a role in the care of children with DCD, and if so, what assessment and management strategies they could employ. Management strategies were divided into either evidence based: such as strength training [11], multidisciplinary engagement [11], activities to promote coordination [11, 13], sensory enhancement aids [11] and non-evidence based strategies as footwear and orthoses [17]. The multiple-choice options were displayed in a random order by the survey software to reduce order effect bias. The final section of questions asked participants about their perceived need for further education on DCD, and in which format they would find most beneficial.

### Procedure

Data collection occurred from May to December 2017. Participants were alerted to the survey via personal or electronic means and provided online informed consent prior to commencing the survey. Survey advertising was repeated using the same channels previously described throughout the data collection period.

The online survey utilised skip logic when participants indicated no familiarity with the term DCD, with the paper-based survey indicating written instructions to ‘skip ahead’. False positive answers (question distractors) were included in questions regarding presentation and management to establish fidelity of responses. The participants were able to withdraw from the survey at any time by closing the browser or failing to complete the paper-based survey and any non-completion was treated as missing data for the remaining non-completed variables. Completing participants were given the option to provide an email address if they wished to receive a copy of the results of the study.

### Data analysis

Statistical analyses were performed using MedCalc Software [25]. Descriptive statistics were used to report participant characteristics and distribution of each variable. Odds ratios were calculated to compare the responses of participants who reported familiarity with DCD, with those who did not report familiarity with DCD for questions regarding management practices. This analysis has been reported as odds ratios with 95% confidence intervals and *p* values, with significance set at *p* < 0.05.

## Results

### Demographics

A total of 365 complete responses were collected, representing approximately 7% of podiatrists registered in Australia at the time of survey. Table 1 details the demographic data collected from respondents.

**Table 1:** Demographic data of participants

Total participants, *N* = 365	N (%) or Mean (SD)
Recency of practice (years practicing)	8.9 (9.0)
Gender (female)	249 (68%)
Original qualification	
Certificate/Advanced certificate	22 (6%)
Diploma/Associate Diploma	44 (12%)
Bachelor/ Bachelor with Honours	266 (73%)
Masters	26 (7%)
Clinical Doctorate	2 (1%)
Other	5 (1%)
Graduating institution	
Charles Sturt University	11 (3%)
Curtin University	8 (2%)
La Trobe University	89 (24%)
Queensland University of Technology	33 (9%)
University of South Australia	131 (36%)
University of Western Australia	5 (1%)
University of Western Sydney	18 (5%)
University of Newcastle	16 (4%)
Auckland University of Technology	5 (1%)
Other (Includes external to Australia and former institutions)	49 (13%)
Year of graduation	
1960–69	1 (0%)
1970–79	7 (2%)
1980–89	45 (12%)
1990–99	96 (26%)
2000–09	97 (27%)
2010–17	119 (33%)
Further tertiary study relating to podiatry (yes)	57 (16%)
Highest qualification (*N* = 54)	
Graduate Certificate	12 (22%)
Graduate Diploma	12 (22%)
Masters by coursework	13 (24%)
Masters by research	3 (6%)
Professional doctorate	1 (12%)
Doctorate by research (PhD)	8 (15%)
Other	5 (9%)
Primary role	
Private Practice	280 (77%)
Public	49 (13%)
Academic (lecturing and/or research)	13 (4%)
Other	23 (6%)
Hours worked per week (mean)	31.8 (11.6)
State/Territory of primary practice	
Qld	40 (11%)
NSW	51 (14%)
ACT	5 (1%)
Vic	109 (30%)
Tas	13 (4%)
SA	135 (37%)
WA	10 (3%)
NT	2 (1%)
Paediatric patient load (%)	
0–25%	309 (85%)
25–50%	50 (14%)
50–75%	2 (1%)
> 75%	4 (1%)

There were 247 (67%) participants who had knowledge of DCD as a diagnosis either by its current name or a historical name. There were 109 (30%) reported familiarity with DCD as a diagnosis. While an additional 134 (37%) participants reported familiarity with alternate terminology that has previously been used to described DCD (Table 2) without having any knowledge of DCD. Dyspraxia was the term most participants reported familiarity with (*n* = 199, 54%), followed by minimal motor dysfunction (*n* = 134, 37%) and clumsy child syndrome (*n* = 97, 27%) (Table 2).

**Table 2:** Familiarity of participants with DCD and alternate terminology

Participant familiarity responses	N (%) of 365 responses
Familiarity with DCD (as terminology):	
Yes	109 (30%)
No	256 (70%)
Familiarity with alternate terminology (indicate as many options as applicable):	
Minimal motor dysfunction	134 (37%)
Minimal cerebral dysfunction	90 (25%)
vDyspraxia/developmental dyspraxia	199 (54%)
DAMP	77 (21%)
Clumsy child syndrome	97 (27%)
Unfamiliar with DCD or its associated terms	118 (33%)

### Presentation of DCD

**Fig. 1:**
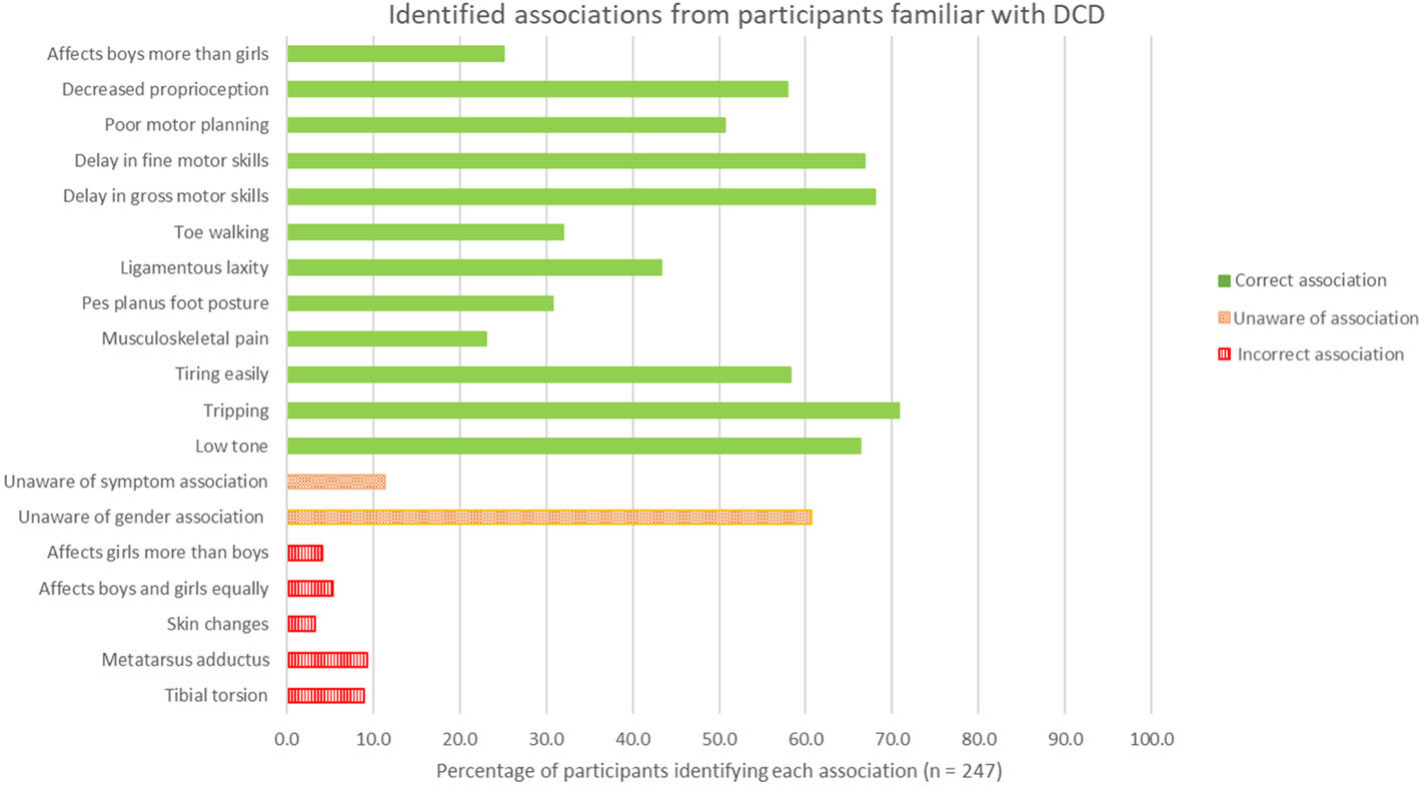
Associations with DCD as identified by podiatrists familiar with the condition

Only participants who reported familiarity with DCD or alternate terminology (*n* = 247, 68%) progressed to questions regarding signs and symptoms (see Fig. 1, Additional file 2). The most commonly reported symptoms were tripping (*n* = 175, 71% of 247 responses), gross motor skill delay (*n* = 168, 68% of 247 responses), low muscle tone (*n* = 164, 66% of 247 responses), and fine motor skill delay (*n* = 165, 67% of 247 responses).

### Assessment and management

**Fig. 2:**
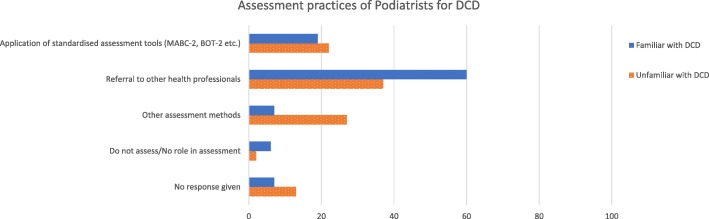
Reported assessment practices for podiatrists familiar and unfamiliar* with DCD as a percentage of participants. *8 responses from participants unfamiliar with DCD were excluded due to a skip logic dysfunction

Participants familiar with DCD (n = 247) were asked what assessment practices they used and previously unfamiliar participants (*n* = 110) were asked what they believed their role in assessment could be (Fig. 2, Additional file 2). Referral to other health professionals for assessment was the most frequently selected response by participants both familiar or unfamiliar with DCD (*n* = 148 (60% of 247) and 40 (37% of 118) respectively). Some podiatrists who were familiar or unfamiliar with DCD reported using standardised clinical assessment tools such as the Motor Assessment Battery for Children 2 (MABC-2) or Bruininks-Oseretsky Test for Motor Proficiency 2 (BOT-2) (*n* = 48 (19% of 247) and 24 (22% of 110) respectively) [26, 27]. Fewer podiatrists who were familiar with DCD reported using other reporting proformas such as the GALLOP, as well as non-standardised assessment methods (*n* = 18, 7% of 247) [28], than those who were unfamiliar (*n* = 30, 27% of 110). Podiatrists familiar with DCD also identified that they did not assess for DCD (*n* = 15, 6% of 247) and a small number of podiatrist unfamiliar with DCD indicated their belief that there was not a role for podiatry in assessment of children with DCD (*n* = 2, 2% of 110), (Fig. 2).

**Fig. 3:**
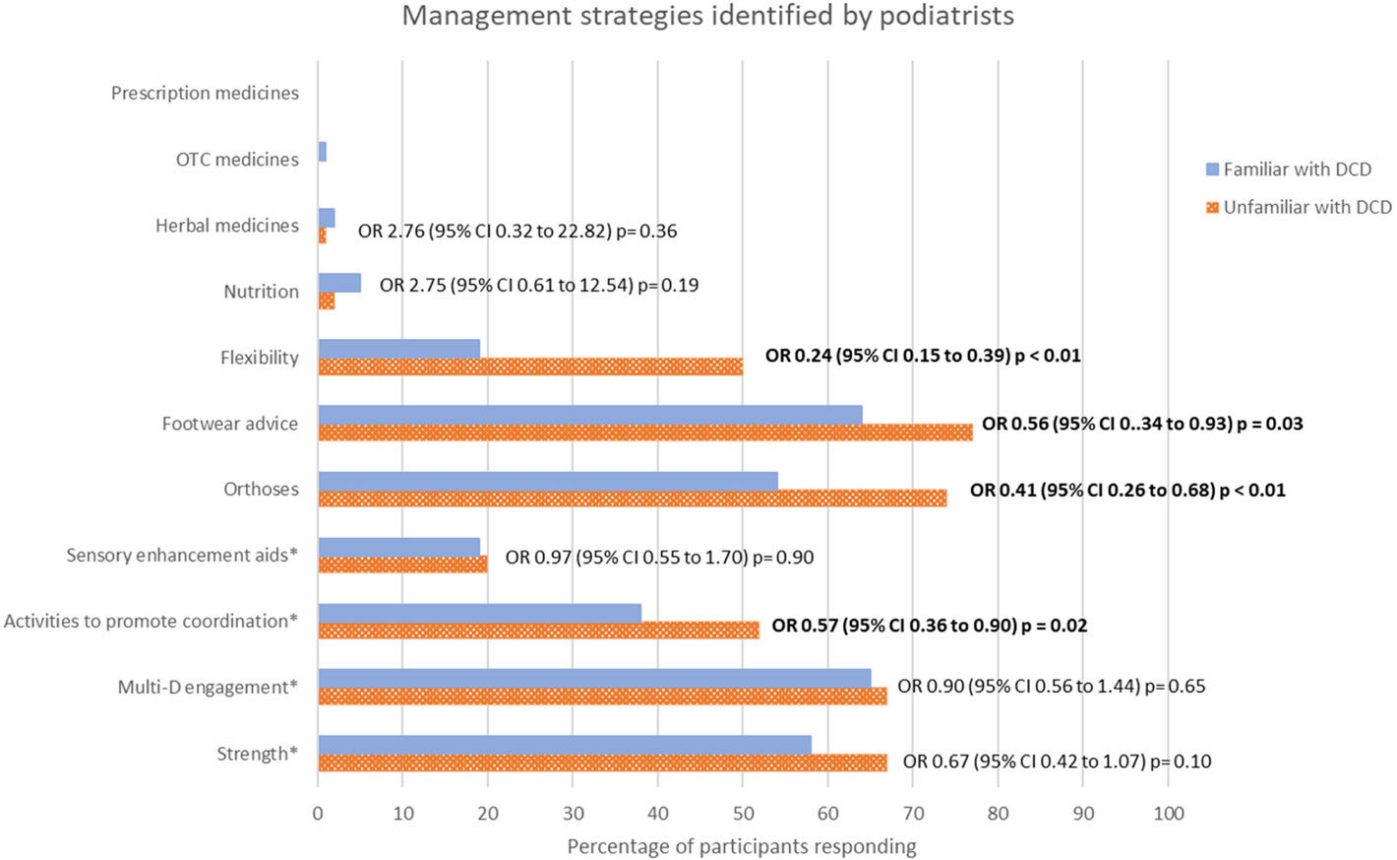
Management strategies identified by podiatrists familiar and unfamiliar^ with DCD as a percentage of participants. *Management strategies for DCD currently supported by evidence. Significant differences indicated by Odds ratio (OR) (95% Confidence Intervals (CI)) and *p* ≤ 0.05 are bolded. ^8 responses were excluded due to a skip logic dysfunction

The majority of respondents in both familiar and unfamiliar groups selected the evidence supported interventions of strength training (*n* = 143, 58% of 247, and *n* = 74, 67% of 110 of familiar and unfamiliar respectively) and multidisciplinary engagement (*n* = 160, 65% and n = 74, 67% of 110 respectively), as well as the non-evidence supported interventions such as footwear advice (*n* = 159, 64% of 247 and *n* = 84, 77% of 110 respectively), and orthoses (*n* = 133, 54% of 247 and *n* = 81, 74% of 110 respectively), (Fig. 3, Additional file 2).

There were significant differences in identifying several management strategies for DCD between those who were familiar and those unfamiliar with the condition (whom had only been given a definition of DCD), (Fig. 3, Additional file 3). More podiatrists unfamiliar with DCD identified management strategies not currently supported by the evidence; increasing flexibility (OR = 0.24 95% CI = 0.15 to 0.39, *p* < 0.001), footwear advice (OR = 0.56, 95% CI = 0.34 to 0.93, *p* = 0.03), and foot orthoses (OR = 0.41, 95% CI = 0.26 to 0.68, *p* = 0.001), meaning those familiar with the condition were less likely to identify these interventions by 76, 43 and 59% respectively. Those unfamiliar with DCD also more frequently identified activities to promote coordination (OR = 0.57, 95% CI = 0.36 to 0.90, *p* = 0.02), meaning those familiar with the condition were 43% less likely to identify this intervention, despite it being supported by evidence.

### Education on DCD

A strong majority of participants (*n* = 304 (83%)) indicated willingness to receive further education on DCD, with the most popular responses being clinical guidelines, factsheets, and online presentations (Table 3).

**Table 3:** Participant views on education around DCD

Participant responses	N (%)
Agreed to receiving education (Yes)	304 (83%)
Most beneficial method:	
Clinical Guidelines	184 (50%)
Online Presentation	148 (41%)
Factsheet	147 (40%)
Video presentation	123 (34%)
In-person presentation	111 (30%)
Not selected a preferred method	24 (7%)
Other (including webinars, online materials or literature)	15 (4%)

## Discussion

This is the first knowledge-based survey of podiatrists about the diagnosis, management and education needs of podiatrists about DCD in children. Approximately a third of respondents reported familiarity with DCD, despite being a prevalent condition in children. Familiarity increased with the addition of alternate or previously used terminology. Similarly, a Canadian study of physicians found only 22% of family doctors were familiar with DCD, which rose to 72% when alternate terminology (dyspraxia and clumsy child syndrome) was used [22]. Developmental coordination disorder has been accepted terminology since the 1994 International Consensus Conference on Children and Clumsiness [23], yet this terminology does not appear to have translated into the common vernacular.

Podiatrists familiar with DCD or alternate DCD terminology were able to correctly recognise signs and symptoms of DCD, with only small proportions of these participants selecting signs with no established link with the condition (such as metatarsus adductus and tibial torsion). Familiarity with the diagnosis but lack of knowledge of the presentation was also identified in a low number of participants. This conceptual recognition of the terminology but lack of actual knowledge is potentially a barrier for informed clinical practice. Additionally, whilst many podiatrists were familiar with DCD (or alternate terms) as a diagnosis, fewer reported conducting the types of assessments that guide treatment options or referrals for additional targeted therapy. Instead, those familiar and unfamiliar with this condition preferred to refer to alternative health professionals; which may highlight a gap in knowledge from training, potentially suggests podiatrists may not be not confident in their scope, or lack of interest in treating this population of children. It also presumably relates to many participants reporting a small paediatric clinical case load. Many standardised assessments such as the BOT-2 [26] have a high cost set up (>$2000 AUD) and require registration to purchase. It is possible that podiatrists with a small paediatric case load would not consider this financially viable and an investment in the types of clinical tools unfeasible or not of interest.

The most frequently reported management strategies identified by podiatrists familiar and unfamiliar with DCD were strength training, orthoses, footwear advice, and multi-disciplinary engagement. Only one study has investigated orthoses and footwear options as a treatment in this population, with results showing non-statistically significant improvement with the use of foot orthoses on spatiotemporal gait parameters [17]. However, the small sample size and lack of significance should lead podiatrists to incorporate these results into practice with a degree of caution. Research has identified motor skill programs which focus on task specific training and incorporating strength training to be an effective intervention to improve motor performance for children with DCD [11, 16, 29, 30]. Activities that promote motor skill and coordination were not chosen as a preferred treatment choice by many of the participants in this study, despite the coordination deficits implicit in the diagnosis and its definition. In particular, those familiar with DCD did not use or promote activities as frequently as those who were unfamiliar with the condition reported they would. This was an interesting result,and may reflect that children with DCD are often treated within a multi-disciplinary team. Those who do see larger proportions of children with DCD may observe that physical activity and coordination activities are provided by the physiotherapist or occupational therapist and not wish to increase family therapy burden. It may also be a lack of confidence in provision due to the challenging nature of the condition. This is an area that may need further exploration with those who routinely see children with DCD.

Despite many participants being unfamiliar with DCD, most reported they would be willing to receive further education about assessment and management of the condition. A further recommendation for education providers is that contemporary education practices be used, and podiatrists appear to preference education strategies similar to other allied health professional [31]. Podiatrists appear to recognise the need for advancement in their knowledge of DCD and are receptive to a number of methods of education.

There are several limitations to this research. This study was exploratory, and its purpose was to gather a basic understanding of the clinical knowledge and practice habits of podiatrists in Australia about DCD in children. For this reason, survey questions were relatively general in nature and captured only basic information.

The limited participant numbers as a percentage of the podiatry population in Australia is also acknowledged. Desired outcomes of management practices were not assessed, and hence participants were not provided an opportunity to give reasoning or justification for their assessment or management choices. This removes the individualised aspect of patient care, for example strength training should be implemented when strength deficits are noted clinically or stretching when there is observed tightness or contracture. Additionally, it did not consider the individualised or pragmatic aspects of management. For example, there is no evidence supporting footwear as a management strategy for DCD, however, a podiatrist may have selected this as they may individualise footwear concept or feature advice for the child who struggles to don shoes or self-manage shoe fixtures. The results of this research should therefore be only considered as preliminary, and not illustrative of any nuance within current podiatric management of children with DCD.

Additional limitations include the sample size and the dual methods of data collection. This sample size represents 7% of the Australian podiatry profession. While this is a low response rate, there was variation observed between and within groups. This limited the ability to postulate the results as representative of the entire profession, however there was still the ability to examine relationships between groups, such as those with or without knowledge of DCD, as these estimates do not necessarily require representative samples [32, 33]. There was potential for an over reporting of knowledge of DCD due to self-selection bias. This was minimised by approaching people at a national conference rather than advertising. These factors have some potential to limit the generalizability of the results to the whole profession. A large cohort of participants were sampled at Melbourne and Adelaide based events leading to an overrepresentation of podiatrists from Victoria and South Australia. In turn, the University of South Australia was overrepresented in the sample as a tertiary institution compared to national demographics as published by the Podiatry Board of Australia. The survey responses were completed both online and in paper form, and those in paper inherently relied on participants to read the skip logic and fully complete. Whilst instructions were given in paper surveys to replicate this skip logic, these were not followed by some participants leading to incomplete or incorrectly completed responses and the exclusion of some data.

## Conclusion

Participating Australian podiatrists were largely unfamiliar with developmental coordination disorder as a diagnosis, although this increased with the introduction of previous terminology. Participants frequently reported referral to other health professionals rather than performing assessments, while recommending common podiatric interventions such as footwear education, orthoses and strength training. Most respondents showed interest in receiving further education on DCD, and given current lack of evidence, future research should focus on determining the role of podiatrists in assessment and management of children with DCD as part of an interprofessional team.

## Additional file

Additional file 1: Survey instrument. (DOCX 1358 kb)

Additional file 2: **Table 2.** a) Associations of DCD as reported by podiatrists familiar with the condition. b) Reported assessment practices for podiatrists familiar and unfamiliar with DCD. (DOCX 15 kb)

Additional file 3: **Table 6.** Differences between management strategies for podiatrists who are and are not familiar with DCD (significant differences are bolded). (DOCX 14 kb)

## Data Availability

All data generated or analysed during this study are included in this published article [and its supplementary information files].

## Abbreviations

BOT-2: Bruininks-Oseretsky Test of Motor Proficiency, Second Edition
DAMP: Disorder of attention and motor planning
DCD: Developmental coordination disorder
DSM-IV or DSM-4: Diagnostic and Statistical Manual of Mental Disorders – 4th edition
DSM-V or DSM-5: Diagnostic and Statistical Manual of Mental Disorders – 5th edition
MABC-2: Motor Assessment Battery for Children, 2nd Edition
