# Supplementary figures and images for: Determining the clinical knowledge and practice of Australian podiatrists on children with developmental coordination disorder: a cross-sectional survey
Source: J Foot Ankle Res. 2019 Aug 13;12:42. doi: 10.1186/s13047-019-0353-y (PMC6693096; doi:10.1186/s13047-019-0353-y)

## Additional File 1: Survey instrument


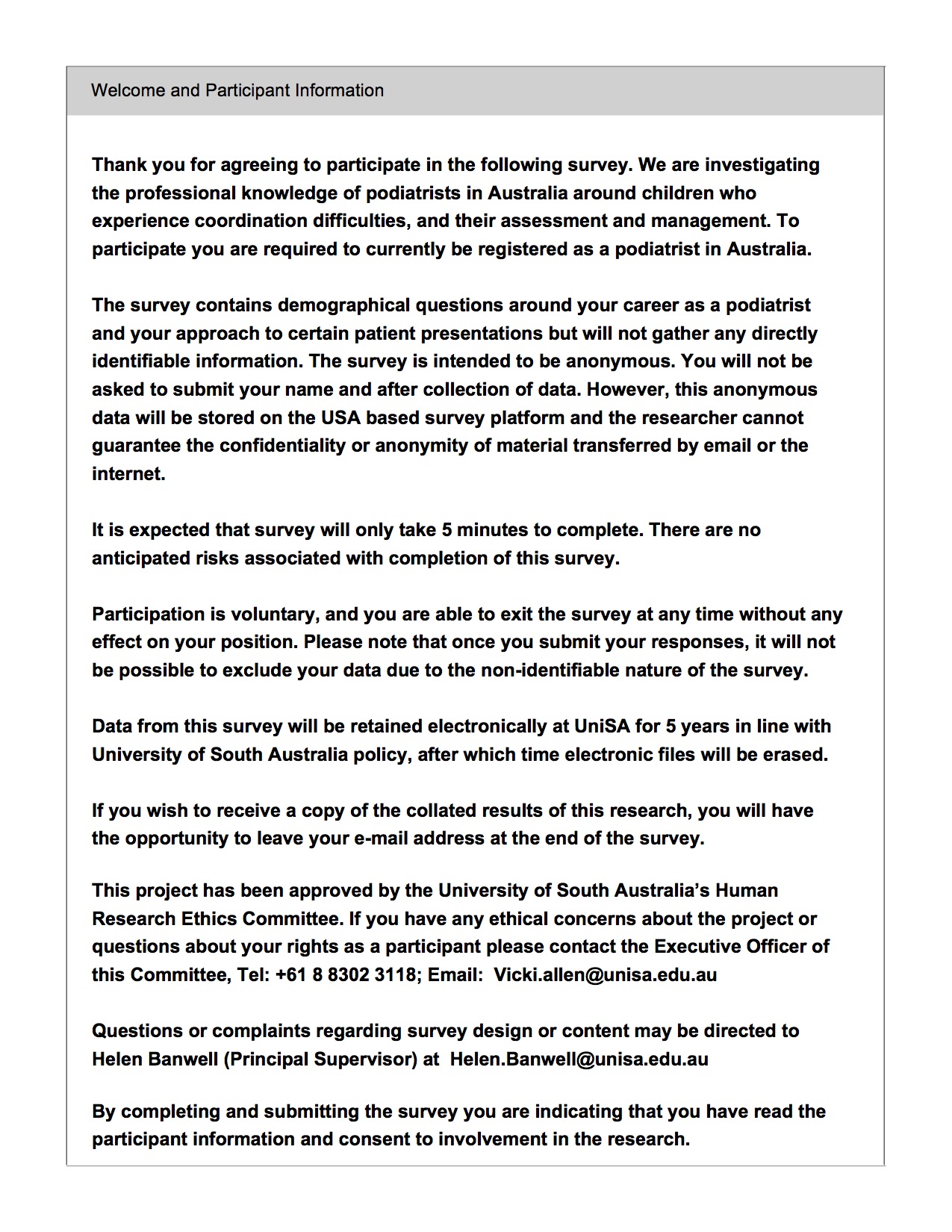


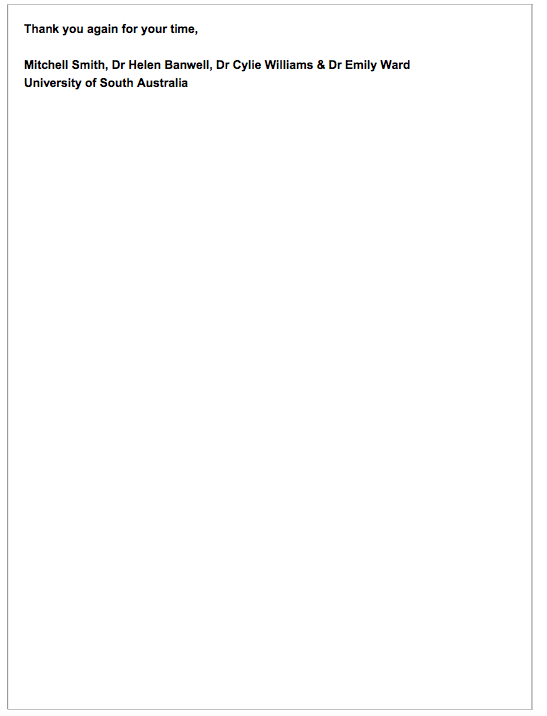


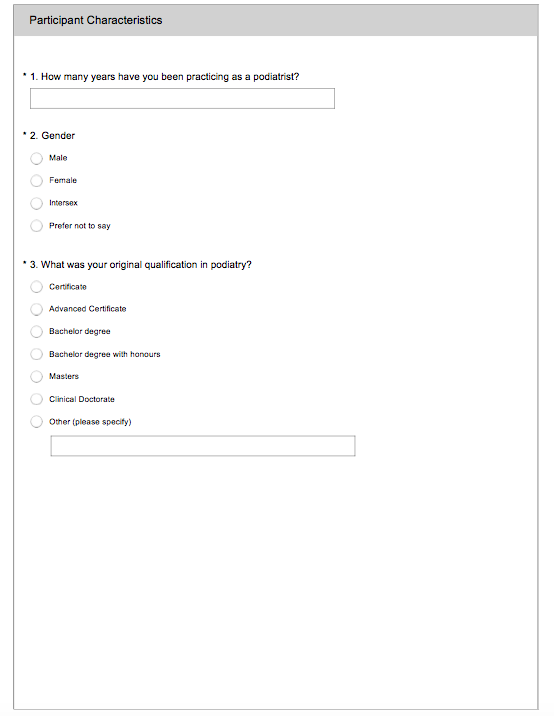


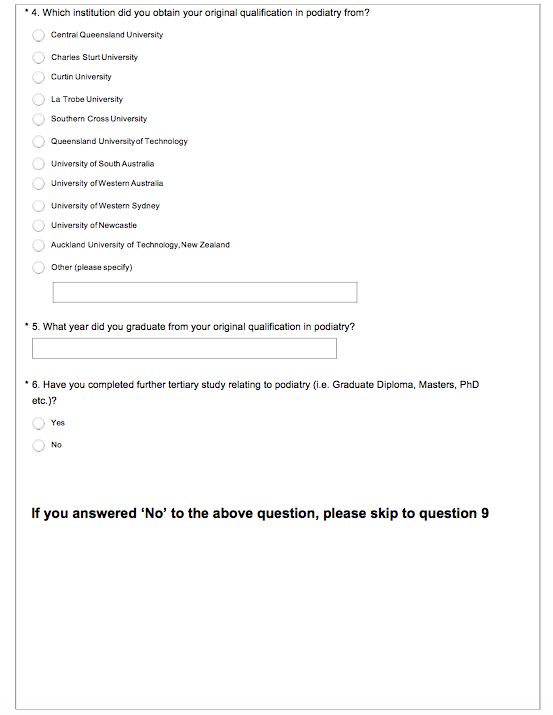


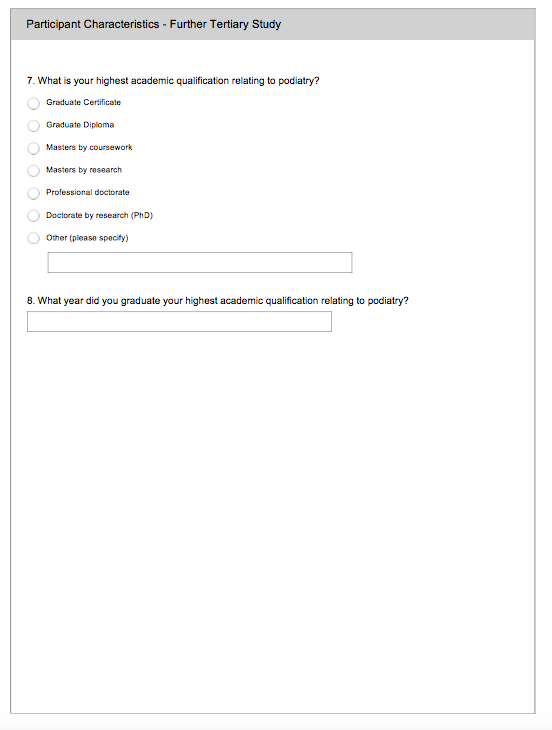


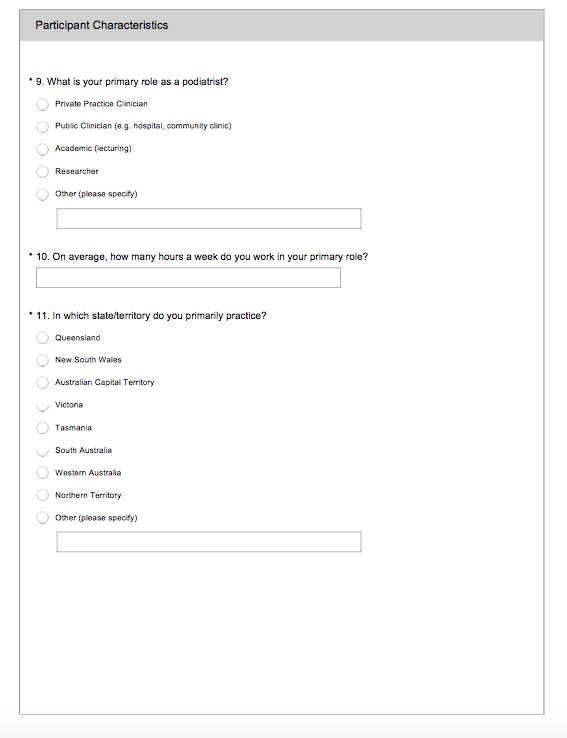


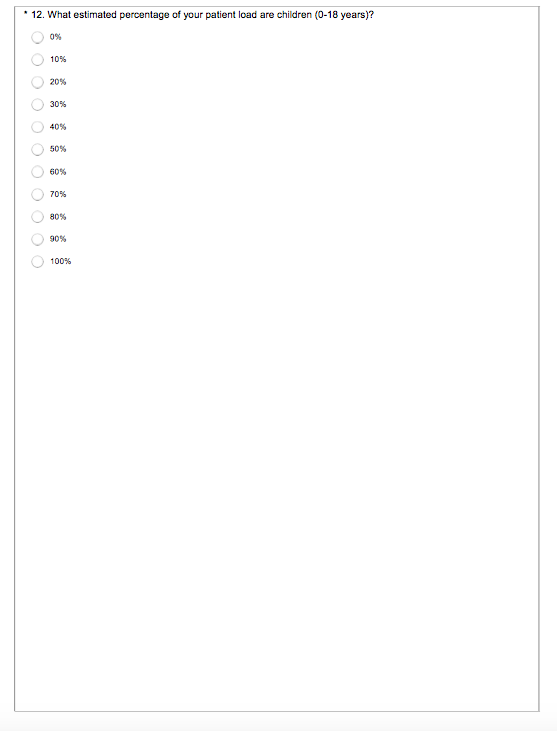


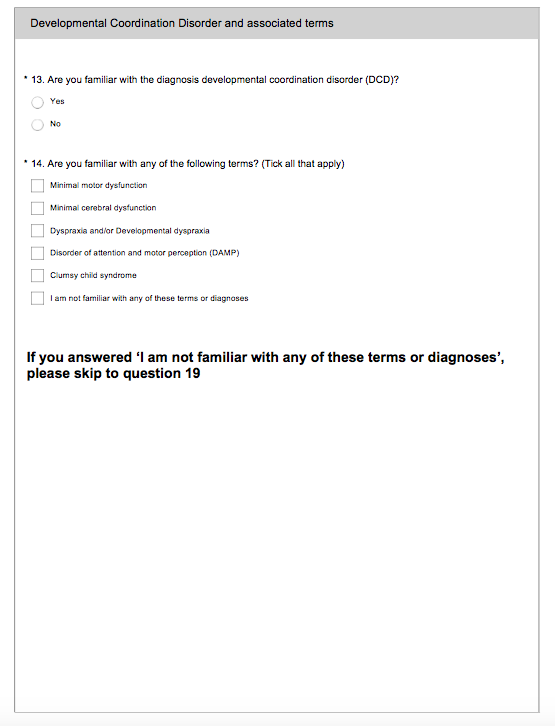


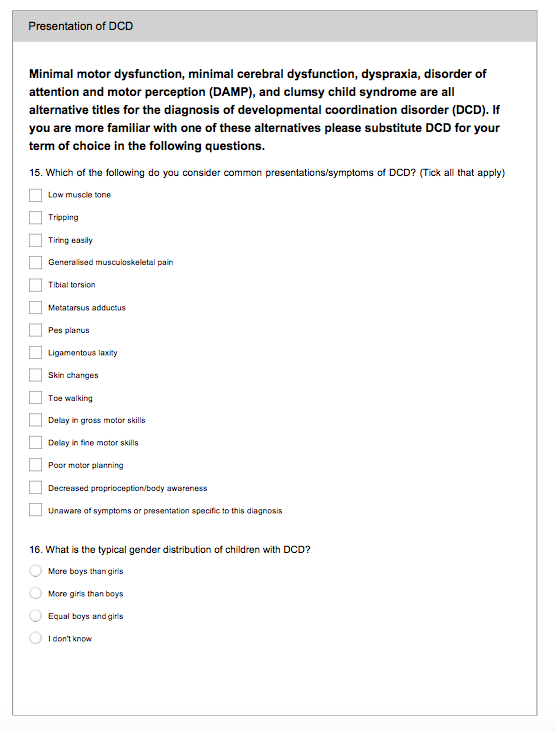


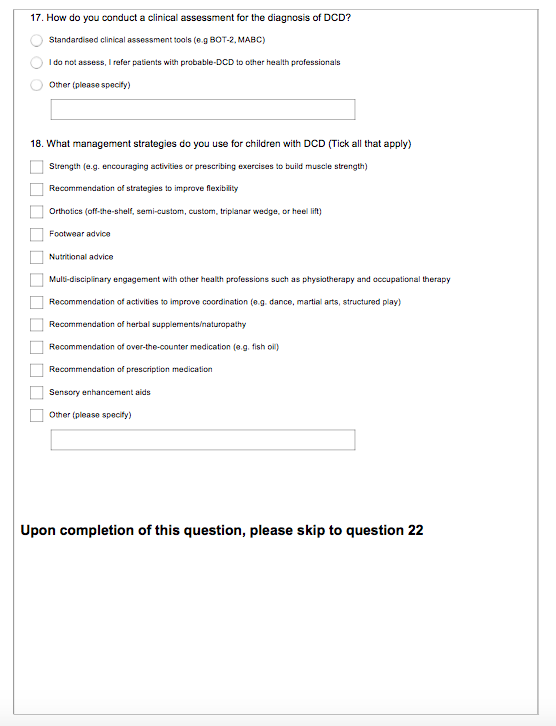


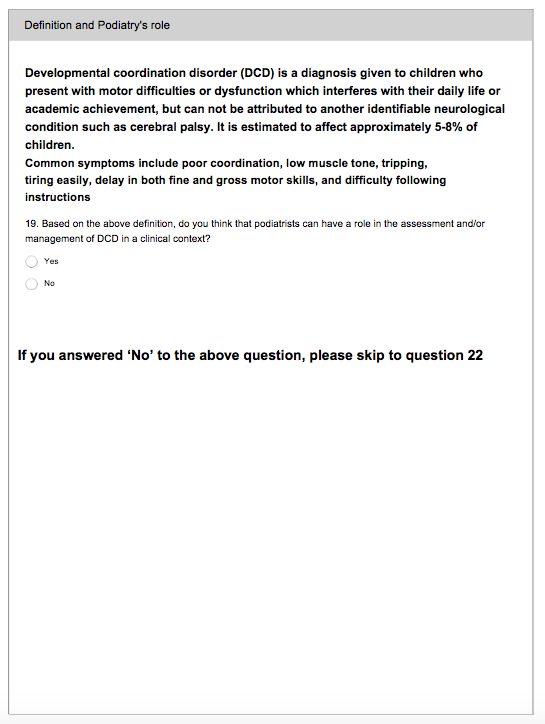


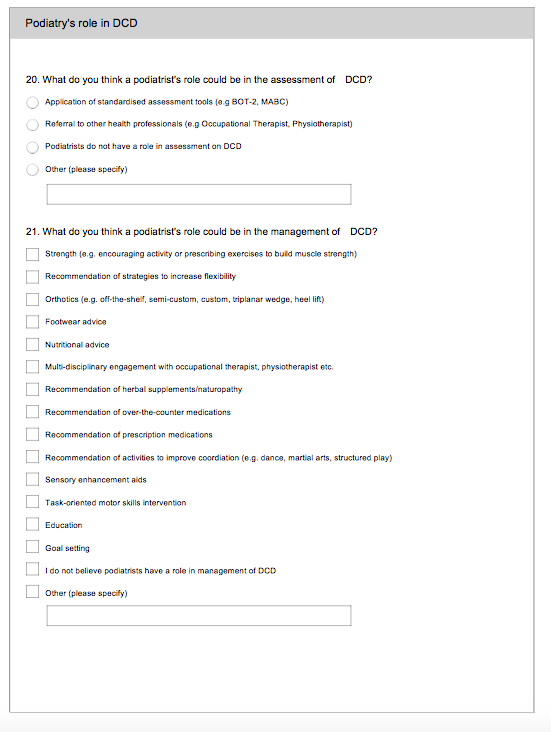


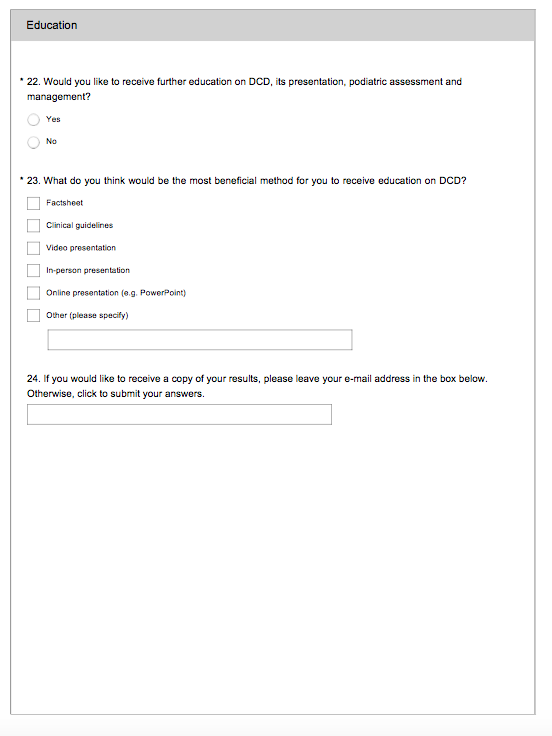


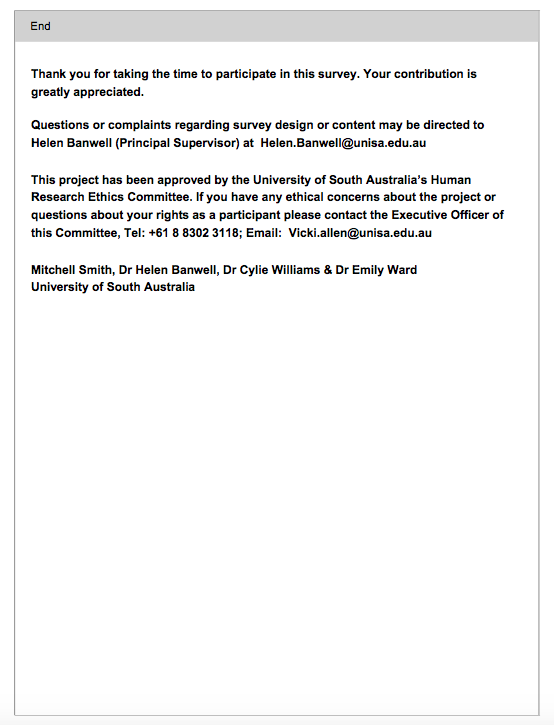

Supplement: Supplementary file 1 — Survey instrument. (DOCX 1358 kb) [file 13047_2019_353_MOESM1_ESM.docx]
