# Supplementary material for: Determining the clinical knowledge and practice of Australian podiatrists on children with developmental coordination disorder: a cross-sectional survey
Source: J Foot Ankle Res. 2019 Aug 13;12:42. doi: 10.1186/s13047-019-0353-y (PMC6693096; doi:10.1186/s13047-019-0353-y)
Supplement: Supplementary file 2 — Table 2. a) Associations of DCD as reported by podiatrists familiar with the condition. b) Reported assessment practices for podiatrists familiar and unfamiliar with DCD. (DOCX 15 kb) [file 13047_2019_353_MOESM2_ESM.docx]

Additional file 2.

Table 2a. Associations of DCD as reported by podiatrists familiar with the condition.

| What do podiatrists consider as common presentations or symptoms of DCD? | N = 247 |
| --- | --- |
| Related signs and symptoms  Low tone  Tripping  Tiring easily  Musculoskeletal pain  Pes planus foot posture  Ligamentous laxity  Toe walking  Delay in gross motor skills  Delay in fine motor skills  Poor motor planning  Decreased proprioception  Unrelated signs and symptoms  Tibial torsion  Metatarsus adductus  Skin changes  Unaware of symptoms related this diagnosis  No response given | 164 (66%)  175 (71%)  144 (58%)  57 (23%)  76 (31%)  107 (43%)  79 (32%)  168 (68%)  165 (67%)  125 (51%)  143 (59%)  22 (9%)  23 (9%)  8 (3%)  28 (11%)  11 (4%) |
| Knowledge of gender distribution  Correct response:  More boys than girls  Incorrect/unknown responses:  More girls than boys  Equal boys and girls  Unaware of gender distribution  No response given | N = 247  62 (25%)  10 (4%)  13 (5%)  150 (61%)  12 (5%) |

Table 2b. Reported assessment practices for podiatrists familiar and unfamiliar with DCD

| Assessment practice | Familiar with DCD  N = 247, n (%) | Unfamiliar with DCD  N = 110*, n (%) |
| --- | --- | --- |
| Application of standardised assessment tools (MABC-2, BOT-2 etc.) | 48 (19%) | 24 (22%) |
| Referral to other health professionals | 148 (60%) | 40 (37%) |
| Non-specific or non-standardised assessment and referral | 18 (7%) | 30 (27%) |
| N/A or Do not assess | 15 (6%) |  |
| No role in assessment |  | 2 (2%) |
| No response given | 18 (7%) | 13 (12%) |

*8 responses were excluded due to a skip logic function resulting in non-completion of assessment and management questioning
