# Supplementary material for: Determining the clinical knowledge and practice of Australian podiatrists on children with developmental coordination disorder: a cross-sectional survey
Source: J Foot Ankle Res. 2019 Aug 13;12:42. doi: 10.1186/s13047-019-0353-y (PMC6693096; doi:10.1186/s13047-019-0353-y)
Supplement: Supplementary file 3 — Table 6. Differences between management strategies for podiatrists who are and are not familiar with DCD (significant differences are bolded). (DOCX 14 kb) [file 13047_2019_353_MOESM3_ESM.docx]

Additional file 3.

Table 6. Differences between management strategies for podiatrists who are and are not familiar with DCD (significant differences are bolded).

|  | Management strategies supported by podiatrists familiar with DCD  n (%) (N = 247) | Management strategies supported by podiatrists not familiar with DCD  n (%) (N = 110*) | Odds Ratio, (95% Confidence Interval), p value |
| --- | --- | --- | --- |
| *Evidence Based Interventions* | | | |
| Strength | 143 (58%) | 74 (67%) | 0.669 (0.417 to 1.072) p=0.095 |
| Multi-D engagement | 160 (65%) | 74 (67%) | 0.895 (0.5557 to 1.440) p=0.647 |
| Activities to promote coordination | 94 (38%) | 57 (52%) | **0.571 (0.363 to 0.899) p=0.016** |
| Sensory enhancement aids | 48 (19%) | 22 (20%) | 0.965 (0.549 to 1.695) p=0.901 |
| *Non-evidence Based Interventions* | | | |
| Orthoses | 133 (54%) | 81 (74%) | **0.412 (0.255 to 0.684) p = 0.001** |
| Footwear advice | 159 (64%) | 84 (77%) | **0.559 (0.335 to 0.932) p=0.026** |
| Flexibility | 48 (19%) | 55 (50%) | **0.241 (0.148 to 0.393) p<0.001** |
| Nutrition | 12 (5%) | 2 (2%) | 2.757 (0.607 to 12.535) p=0.189 |
| Herbal medicines | 6 (2%) | 1 (1%) | 2.714 (0.323 o 22.815) p=0.358 |
| OTC medicines | 3 (1%) | 0 (0%) | N/A |
| Prescription medicines | 1 (0%) | 0 (0%) | N/A |

*8 responses were excluded due to a skip logic function resulting in non-completion of assessment and management questioning
